# Transient CRISPR-Cas Treatment Can Prevent Reactivation of HIV-1 Replication in a Latently Infected T-Cell Line

**DOI:** 10.3390/v13122461

**Published:** 2021-12-08

**Authors:** Ye Liu, Rienk E. Jeeninga, Bep Klaver, Ben Berkhout, Atze T. Das

**Affiliations:** Amsterdam UMC, Laboratory of Experimental Virology, Department of Medical Microbiology and Infection Prevention, University of Amsterdam, 1105AZ Amsterdam, The Netherlands; y.liu2@amsterdamumc.nl (Y.L.); jeeninga@viroclinics.com (R.E.J.); g.p.klaver@amsterdamumc.nl (B.K.); b.berkhout@amsterdamumc.nl (B.B.)

**Keywords:** HIV, CRISPR-Cas, Cas9, Cas12a, transient, RNP

## Abstract

Novel therapeutic strategies aiming at the permanent inactivation of the HIV-1 reservoir in infected individuals are currently being explored, including approaches based on CRISPR-Cas gene editing. Extinction of all infectious HIV provirus in infected T-cell cultures was previously achieved when cells were transduced with lentiviral vectors for the stable expression of CRISPR-Cas9 or Cas12a systems targeting HIV DNA. Because lentiviral transduction and long-term CRISPR-Cas activity are less suitable for in vivo application of this antiviral strategy, we investigated whether HIV can also be completely inactivated by transient CRISPR-Cas activity. Latently infected SupT1 T-cells were repeatedly transfected with different Cas9 and Cas12a mRNA/protein sources in combination with dual gRNAs/crRNAs targeting highly conserved viral sequences. Upon repeated Cas9 protein treatment, viral replication could no longer be reactivated. We demonstrate that this was due to complete mutational inactivation of the proviral DNA, mostly through mutations at the target sites, but also through excision or inversion of the viral DNA fragment between the two target sites. These results demonstrate that repeated transient CRISPR-Cas treatment of a latently infected T-cell culture can lead to the permanent inactivation of HIV replication, indicating that transient CRISPR-Cas delivery methods can be considered for in vivo application.

## 1. Introduction

Since the discovery of HIV as the causative agent for AIDS almost 40 years ago, extensive research has resulted in antiretroviral therapy (ART) with a combination of drugs that can reduce the viral load in HIV-infected patients to undetectable levels, but unfortunately, does not permanently cure the patient. These drugs target essential processes in the viral replication cycle, such as reverse transcription of the viral RNA genome into a DNA copy and integration of this DNA in the cellular genome, but do not inactivate the proviral DNA in infected reservoir cells [1,2,3]. As the viral load rebounds when ART is halted, HIV-infected individuals need lifelong therapy with potential toxicity, and there is the continuous risk of the development of drug resistance [4,5]. Therefore, novel therapeutic strategies aiming at the permanent inactivation of the viral reservoir are explored, including targeting the integrated provirus with CRISPR-Cas gene-editing systems (reviewed in [6,7]).

The CRISPR-Cas9 system originating from *Streptococcus pyogenes* has been most frequently applied in such anti-HIV studies. In this system, a CRISPR RNA (crRNA) with a 20-nt ‘spacer’ sequence combines with a trans-activating crRNA (tracrRNA) and guides the Cas9 endonuclease to a complementary ‘protospacer’ target sequence in double-stranded DNA [8]. A single guide RNA (gRNA) in which the crRNA and tracrRNA sequences are fused is frequently used in gene-editing applications. Besides sequence complementarity between the crRNA or gRNA and the target DNA, Cas9 binding requires the presence of a short sequence motif (NGG for SpCas9) immediately adjacent to the protospacer sequence (‘protospacer adjacent motif’; PAM). Cas9 cleavage results in double-stranded, blunt-ended DNA breaks that can be repaired by cellular DNA repair mechanisms, in particular non-homologous end-joining (NHEJ) and microhomology-mediated end-joining (MMEJ) [9,10]. These repair pathways will introduce mutations at the cleavage site, in particular nucleotide deletions and insertions (indels) but also nucleotide substitutions, which can inactivate the targeted gene function. We and others demonstrated that HIV replication could be inhibited in vitro by harnessing the host cells with Cas9 and a single gRNA targeting the proviral DNA [11,12,13,14]. However, the virus frequently escaped from this inhibition, and sequence analysis revealed that the resistant HIV variants carried mutations at the target site that prevent gRNA binding but that do not inactivate virus replication. Thus, Cas9 cleavage and subsequent cellular DNA repair do not only cause inhibition of virus replication but also facilitate the generation of virus escape variants [15]. When the cells were harnessed with Cas9 and two gRNAs that target different highly conserved and essential domains in the HIV genome, more durable virus inhibition was obtained [16,17]. Such dual-gRNA targeting of the proviral DNA resulted not only in mutations at the target sites but also, albeit less frequently, in excision and rarely inversion of the fragment between the target sites [18]. Some gRNA pairs completely prevented virus escape and resulted in complete and permanent virus inactivation [16], which demonstrated that HIV-infected T-cell cultures could be cured in vitro.

More recently, the CRISPR-Cas12a (Cpf1) system from the *Lachnospiraceae bacterium* was used to target HIV because this system seems to have several advantages over the SpCas9 system [19,20], such as increased target sequence specificity and reduced off-targeting potential, a smaller gene size (3.7-kb for LbCas12a; 4.1-kb for SpCas9) and the 43-nt crRNA does not require a tracrRNA for its guiding function and is smaller than the ∼100-nt Cas9 gRNA [21,22,23]. The size and crRNA characteristics may be particularly beneficial when using viral vectors with a limited capacity to store genetic information for gene transfer [24]. LbCas12a requires a different PAM sequence (TTTN), which allows targeting of another set of HIV sequences. Furthermore, Cas12a cleaves the DNA strands asymmetrically, which results in sticky ends with a 5′ overhang. When cells were harnessed with Cas12a and a single antiviral crRNA, we observed potent inhibition of virus replication, like we observed with Cas9 and a single guide RNA [19]. We also frequently observed viral escape from this inhibition due to mutations at the target site. However, this was not seen in all cultures. We observed permanent HIV inactivation with some single crRNAs, which suggests that the LbCas12a system has superior antiviral activity compared to the Cas9 system that required two gRNAs for complete HIV inactivation. Possibly, the different characteristics of the Cas12a target site, with an asymmetric cleavage site that is located more distal from the PAM and does not overlap the ‘Seed’ sequence that is most important for crRNA binding, result in more extensive target site mutations, which will reduce the ability of HIV to escape [19].

Although these studies demonstrate that HIV-infected cells can be cured in vitro with the CRISPR-Cas antivirals, several hurdles need to be overcome before this strategy can be applied in humans. One of the main issues is the delivery of the CRISPR-Cas antivirals to all HIV-infected cells in vivo. We previously used lentiviral vectors with Cas9 and crRNA expression cassettes to transduce T cells in vitro, which resulted in the continuous presence of the antivirals in the stably transduced cells. However, lentiviral transduction and continuous CRISPR-Cas activity seem less suitable for in vivo application of this antiviral strategy because of possible insertional mutagenesis of the vector and off-target Cas cleavage effects. Several other studies used transient methods for the delivery of anti-HIV CRISPR-Cas reagents, such as plasmid transfection or virus-like vesicles [6,25]. In most of these studies, however, non-replicating HIV-derived constructs were targeted instead of replication-competent HIV genomes, while other studies did not test for complete and permanent inactivation of replication-competent virus. Therefore, we set out to test whether cells can also be cured of replication-competent HIV by a transient CRISPR-Cas treatment. T cells latently infected with a replication-competent HIV strain were repeatedly transfected with different CRISPR Cas reagents, i.e., Cas mRNA or protein in combination with dual antiviral crRNAs, after which the viral capacity for gene expression and replication was analyzed.

## 2. Materials and Methods

Virus and cell culture. The HIV-rtTA-GFP virus is a derivative of the HIV-1 LAI isolate [26], in which (1) the Tat-TAR axis of transcription activation is inactivated through mutations in the Tat gene and the TAR hairpin sequence [27,28], (2) the optimized rtTA and tet operator components of the doxycycline-inducible Tet-On regulatory mechanism [29] are inserted at the site of the Nef gene and in the U3 promoter region, respectively, and (3) the internal ribosome entry site (IRES) of the *encephalomyocarditis virus* (EMCV) and the enhanced GFP gene are inserted between the rtTA and 3’ LTR sequences. The SupT1 T-cell line [30] was obtained through the NIH HIV Reagent Program (Available online: www.hivreagentprogram.org; ARP-100 accessed on 2 November 2021). SupT1 cells with multiple copies of the HIV-rtTA-GFP proviral genome were generated by infection of SupT1 cells with HIV-rtTA-GFP virus (to be described elsewhere). A clonal cell line with high GFP expression in the presence of dox and no expression without dox was selected. Cells were grown in advanced RPMI1640 medium (GIBCO, Life Technologies, Bleiswijk, Netherlands) with 1% fetal bovine serum (FBS), 15 U/mL of penicillin, 15 U/mL of streptomycin mix and 1% L-glutamine in a humidified incubator at 37 °C and 5% CO_2_. When indicated, 1 µg/mL dox (Sigma D-9891; Sigma-Aldrich, Saint Louis, MO, USA) was added.

Nucleofection of CRISPR-Cas reagents. Chemically synthesized Cas9 tracrRNA (IDT 1072534), Cas9 crRNAs (Alt-R CRISPR-Cas9 crRNA XT) targeting *gag* (5′-GTTAAAAGAGACCATCAATG-3′ for gGag1) and *tat*/*rev* (5′-TCTCCGCTTCTTCCTGCCAT-3′ for gTatRev), and Cas12a crRNAs (Alt-R CRISPR-Cpf1 crRNA) targeting *gag* (5′-TTCCTGAAGGGTACTAGTAGTTC-3′; crGag1) and *tat*/*rev* (5′-ATAGAGAAACTTGATGAGTCTGA-3′; crTatRev) were obtained from IDT (Leuven, Belgium) and reconstituted in Nuclease-Free Duplex Buffer (IDT). Cas9 crRNA:tracrRNA duplexes (gRNAs) were generated by mixing the 200 μM crRNA with 200 μM tracrRNA (1:1), heating at 95 °C for 5 min, and slow cooling down to room temperature. The duplex was stored at −20 °C. For nucleofection, 1 μL SpCas9 mRNA (1 µg/ul; GeneArt™ CRISPR Nuclease mRNA; Invitrogen A29378, Life Technologies, Bleiswijk, Netherlands) or 2 μL SpCas9 protein (5 µg/µL; TrueCut Cas9 Protein v2 NLS; Invitrogen A36499 Waltham, MA, USA) was mixed with 1 μL 100 µM gRNA duplex, and 1 μL *Acidaminococcus* sp. *BV3L6* Cas12a protein (10 µg/µL; Alt-R A.s. Cas12a (Cpf1) Ultra nuclease; IDT, Leuven, Belgium; 10001273) was mixed with 1 μL 100 µM crRNA. The volume was completed with RNase-free water until 5 µL. SupT1-HIV-rtTA-GFP cells were centrifuged for 5 min at 300× *g* and washed with PBS. For each nucleofection, 3.5 × 10^5^ cells were resuspended in 20 μL SF Cell Line Nucleofector solution (4D-Nucleofector X Kit S; Lonza, Verviers, Belgium; V4XC-2032) and mixed with 5 µL CRISPR-Cas reagents or water (no-Cas control). Nucleofection was performed with the 4D-Nucleofector^TM^ X Unit (Lonza, Verviers, Belgium), using the CA-137 program. Cells were incubated for 10 min at room temperature, mixed with 80 μL pre-warmed medium and transferred to a 1 cm^2^ well (48 well plate) filled with 200 µL pre-warmed medium. Cells were cultured at 37 °C and 5% CO_2_ and split 1 to 10 twice a week.

Gene expression and proviral DNA analysis. The CA-p24 level in the culture supernatant was monitored by ELISA as described previously [31]. Bright field and fluorescence microscopy were performed with a fluorescence microscope (EVOS FL, AMG, Thermo Fisher Scientific, Landsmeer, The Netherlands), using the GFP filter setting. In order to analyze the proviral DNA in CRISPR-Cas treated cells, cells were harvested by centrifugation, and intracellular DNA, including the integrated proviral DNA, was isolated with the DNeasy Blood and Tissue Kit (QIAGEN, Venlo, The Netherlands) and quantitated by a Spectrophotometer (NanoDrop 1000, Thermo Fisher Scientific, Landsmeer, The Netherlands). For qPCR analysis, 40 ng DNA was mixed with 1× LC480 MasterMix (Roche Mannheim, Germany), 900 nM forward primer a (gGag1-forward ACCTAGAACTTTAAATGCATGG), 900 nM reverse primer b (gGag3-reverse CGGTCTACATAGTCTCTAAAGG), c (gTatRev-forward AGCCAGTAGATCCTAGAC) or d (gTatRev-reverse CTACTACTAATGCTGCTATTGC) and 200 nM 6-carboxyfluorescein (6-FAM)-labelled gGag1 probe (CCCATGTTTTCAGCATTATCAGAAGGAGCC) in a total volume of 20 µL and analyzed by the LightCycler 480 (Roche, Mannheim, Germany) [18]. As a template for the qPCR standard curves, wild-type and modified (excision and inversion) proviral DNA fragments were used that had been generated by PCR using the same primer pairs as in the qPCR and subsequent TA-cloning in pCRII-TOPO. The full-length qPCR product (obtained with primer pair a + b) was TA cloned into pCRII-TOPO, followed by BDT sequencing of the gGag1 and crGag1 target regions.

## 3. Results

**Figure 1:**
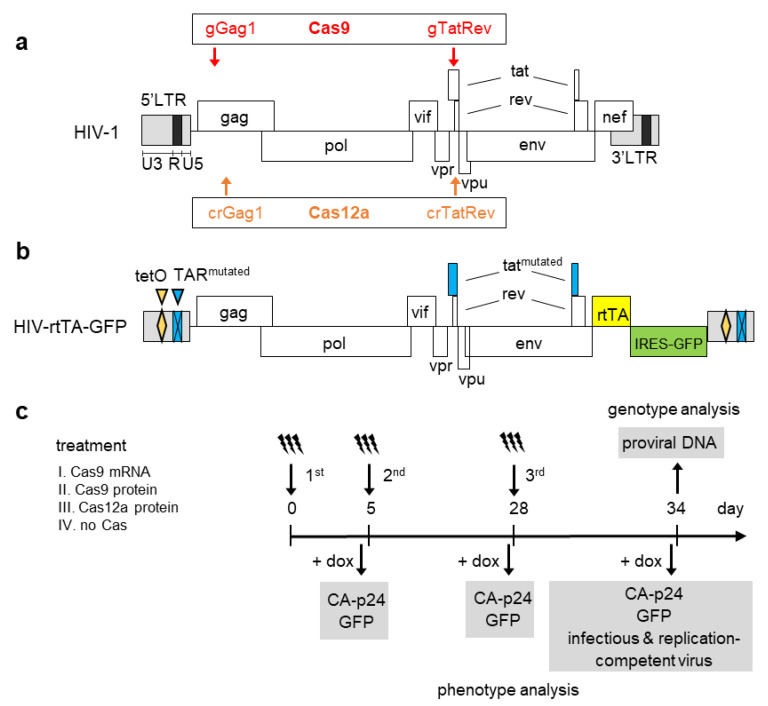
HIV proviral DNA structure and experimental setup. (**a**) The HIV-1 proviral DNA with the position of the gRNA and crRNA target sites indicated (red and orange arrows, respectively). (**b**) The HIV-rtTA-GFP proviral DNA. In this virus, the Tat-TAR axis of transcription activation is inactivated through mutations in both the TAR and Tat sequences (indicated in blue). The reverse tetracycline-controlled transcriptional activator (rtTA) gene is inserted at the site of the Nef gene, and tet operator (tetO) sequences are inserted in the U3 domains (indicated in yellow). Furthermore, an internal ribosome entry site (IRES) and enhanced GFP gene are inserted between the rtTA and 3’LTR sequences (indicated in green). These mutations do not affect the gRNA and crRNA target sites. Upon administration of the tetracycline-derivative doxycycline (dox), rtTA binds to the tetO elements in the U3 promoter region and activates viral transcription. (**c**) Experimental setup. SupT1 cells with integrated HIV-rtTA-GFP proviruses cultured in the absence of dox were repeatedly treated with different CRISPR-Cas reagents. Cas9 mRNA (I) and Cas9 protein (II) were combined with gGag1 plus gTatRev. Cas12a protein (III) was combined with crGag1 plus crTatRev. Control cells (IV) were mock-treated. The treated cells were tested for their capacity to produce virus-encoded proteins (CA-p24 and GFP) and infectious replication-competent viruses upon the administration of dox (+dox). The proviral DNA in the treated cells was analyzed by qPCR and sequencing to characterize the Cas-induced mutations in the proviral DNA (genotype analysis).

We previously demonstrated efficient inhibition of HIV in infected T-cell cultures upon transduction with lentiviral vectors expressing CRISPR Cas9 or Cas12a systems targeting the viral genome [16,18,19,32]. Selection of the transduced cells yielded a population of cells that stably expressed the CRISPR antivirals, which resulted in permanent inactivation of the virus when cells were harnessed with gRNAs or crRNAs targeting highly conserved viral sequences. In order to investigate whether HIV can also be efficiently inhibited and inactivated by transient CRISPR Cas activity, we treated latently infected cells with different commercially available Cas sources, Cas9 mRNA, Cas9 protein or Cas12a protein. Cas9 mRNA and protein were combined with crRNA-tracrRNA complexes, further referred to as gRNAs, targeting highly conserved sequences in the Gag (gGag1) and the overlapping Tat and Rev ORFs (gTatRev; Figure 1a). Cas12a protein was combined with crRNAs targeting the same regions (crGag1 and crTatRev), albeit at a slightly different position because of a different PAM requirement. Targeting of these positions with gRNAs/crRNAs was previously shown to be highly effective in our ‘stable’ CRISPR Cas anti-HIV experiments [11,16,19]. We used SupT1 T cells infected with a doxycycline(dox)-inducible and GFP-expressing HIV-1 variant (HIV-rtTA-GFP, Figure 1b) as a latency model. Gene expression of this virus is off in the absence of dox and can be switched on by dox-administration [33,34]. As a consequence, the production of the viral proteins and GFP, and virus replication are strictly dox-dependent. The experimental setup is shown in Figure 1c. Cells were cultured in the absence of dox and repeatedly transfected (at day 1, 5 and 28) with (I) Cas9 mRNA combined with gGag1 and gTatRev RNAs, (II) Cas9 protein combined with gGag1 and gTatRev RNAs, or (III) Cas12a protein combined with crGag1 and crTatRev RNAs. Control cells (IV) were mock-transfected. After each transfection, inactivation of the proviral gene expression was monitored by culturing a sample of the treated cells with dox, with subsequent analysis of CA-p24 and GFP production (phenotype analysis). In addition, to determine whether the CRISPR-Cas treated cells were still able to produce infectious virus, the culture supernatant of these dox-induced cells was added to regular SupT1 cells, after which these cells were cultured with dox and monitored for a spreading infection of HIV-rtTA-GFP. Finally, the integrated viral DNA was analyzed by qPCR and sequencing to determine the frequency and pattern (mutation, excision, inversion) of provirus inactivation (genotype analysis).

**Figure 2:**
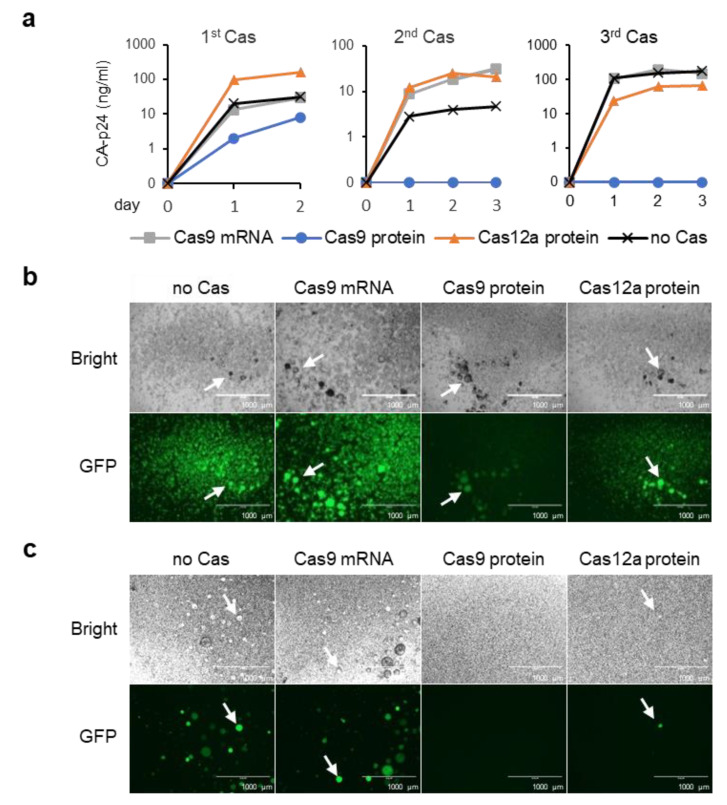
Phenotype analysis of CRISPR-Cas treated HIV-rtTA-GFP infected cells. (**a**) After the 1st, 2nd and 3rd Cas treatment, SupT1-HIV-rtTA-GFP cells were cultured with dox, and HIV capsid protein (CA-p24) was measured in the culture supernatant. (**b**) Control (no Cas) and CRISPR-Cas treated cells (after the 3rd Cas treatment) were cultured with dox for 3 days, and virus-induced syncytia formation and GFP production was analyzed by bright field and fluorescence microscopy. Arrows indicate virus-induced syncytia. (**c**) To analyze the capacity of the control (no Cas) and CRISPR-Cas treated SupT1-HIV-rtTA-GFP cells to produce infectious virus, regular SupT1 cells were infected with 200 µL cell-free culture supernatant from the dox-activated cells shown in panel b. Infected cells were cultured with dox, and virus-induced syncytia formation and GFP production were analyzed by bright field and fluorescence microscopy after 6 days.

Upon the administration of dox to the control (untreated) cells, the CA-p24 level in the culture medium rapidly increased, which reflects dox-induced viral gene expression and replication (Figure 2a). When cells were treated with Cas9 protein, the CA-p24 level after the first treatment was reduced compared to the control cells, whereas no CA-p24 was detectable after the second and third treatment. In contrast, the CA-p24 level in the Cas9 mRNA treated cell cultures remained high after the 1st, 2nd and 3rd treatment, indicating that repeated Cas9 mRNA treatment did not reduce viral gene expression. Cell cultures treated once or twice with Cas12a protein also did not demonstrate a reduced CA-p24 level, whereas a slightly reduced CA-p24 production compared to the control cells was observed after the 3rd treatment. Analysis of GFP expression by fluorescence microscopy revealed a similar pattern (Figure 2b and Supplementary Materials Figure S1). High GFP production was observed in the control cells and the cells that were treated up to three times with Cas9 mRNA. GFP production of the Cas9 protein-treated cells was slightly reduced after the first treatment and greatly reduced after the second and third treatment. GFP production of the Cas12a-treated cells was similar to that of the control cells after the 1st and 2nd treatment and slightly reduced after the 3rd treatment. These CA-p24 and GFP expression analyses indicate that Cas9 protein treatment inactivated viral gene expression and replication more effectively than Cas12a protein treatment, whereas Cas9 mRNA treatment had no effect. In fact, triple Cas9 protein treatment reduced CA-p24 production below the detection level, and only a very low level of GFP fluorescence was detectable.

After the 3rd CRISPR-Cas treatment, we also tested the capacity of the cells to produce infectious, replication-competent viruses. Cells were cultured with dox to activate virus production, and after 3 days, a relatively large volume of the culture medium (200 µL) was used to infect regular SupT1 T cells. The infected cells were cultured with dox and virus replication was monitored by fluorescence microscopy. When the culture medium of Cas9 mRNA-treated cells or mock-treated control cells was tested, we observed the formation of multiple virus-induced syncytia and high GFP expression at 6 days after infection (Figure 2c), which indicates that Cas9 mRNA treatment did not affect the production of infectious virus. In contrast, when testing the culture medium of the Cas9 protein-treated cells, GFP expression and syncytia formation were not detectable at day 6 (Figure 2c) and also not upon prolonged culturing for up to 30 days (data not shown), which indicates that triple Cas9 protein treatment completely abolished production of infectious virus. When the culture medium of the Cas12a protein-treated cells was tested, few GFP-expressing syncytia were detectable, indicating that triple Cas12a protein treatment reduced, but not completely abolished, the production of infectious virus.

**Figure 3:**
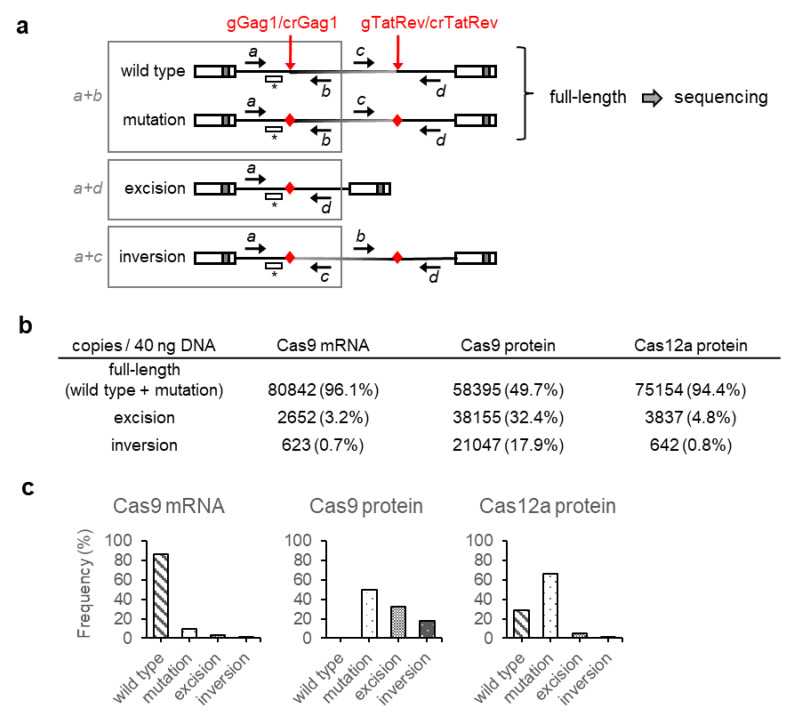
Genotype analysis of CRISPR-Cas treated HIV-rtTA-GFP infected cells. (**a**) Quantitative PCR analysis of proviral DNA products resulting from CRISPR-Cas induced cleavage. A schematic of the proviral DNA is shown, with the position of the gRNA/crRNA target sites indicated by red arrows, the position of qPCR primers a-d indicated by black arrows, and the target site of the FAM-labelled qPCR probe indicated by *. Primer combination a + b detects full-length fragments with either a wild-type or mutated sequence; a + d detects excision products and a + c detects inversion products. (**b**) Intracellular (provirus-containing) DNA isolated from the CRISPR-Cas treated SupT1-HIV-rtTA-GFP cells (after the 3rd treatment) was analyzed by qPCR to quantitate full-length, excision and inversion proviral DNA products. (**c**) The qPCR data from panel b were combined with the wild-type to mutant ratio determined in Figure 4 to calculate the frequency of the wild-type, mutation, excision and inversion products.

We previously demonstrated that CRISPR-Cas9 attack of proviral DNA with dual gRNAs could result in mutation of the gRNA target sites, excision of the fragment between the target sites, or inversion of this DNA fragment [16,18]. To analyze the proviral DNA modifications resulting from the different transient CRISPR-Cas treatments, intracellular DNA was isolated after the 3rd Cas treatment, and the proviral DNA was analyzed by qPCR using different primer combinations around the gGag1 and crGag1 target sites to quantify the full-length, excision and inversion products (Figure 3a). As the full-length PCR fragments can have either a wild-type or mutated target site, we sequenced these DNA products to determine the type of lesion and the mutational frequency.

**Figure 4:**
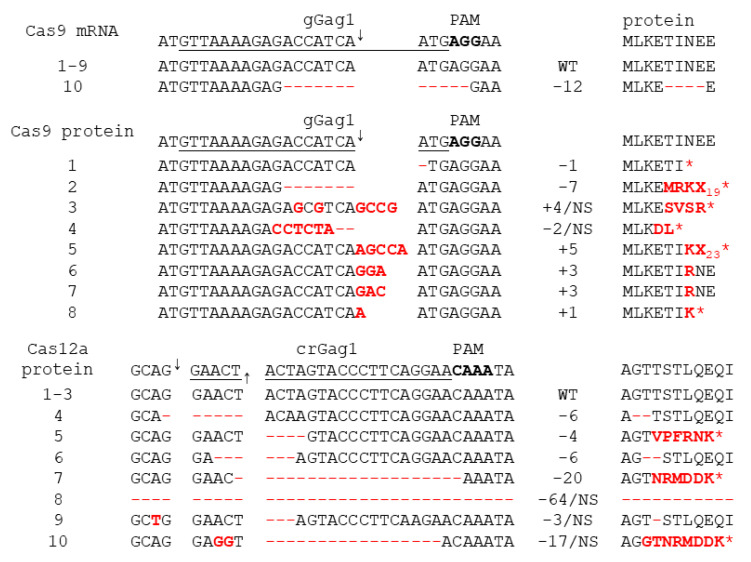
Mutations at the gGag1 and crGag1 target sites after the 3rd CRISPR-Cas treatment. The full-length PCR products obtained with primer combination a + b (as described in Figure 3a–b) were TA cloned, and the gGag1/crGag1 region in 8-10 cloned fragments was sequenced. Sequences were aligned to the wild-type HIV sequence, with the gGag1 and crGag1 target sites underlined, the PAM sequence in bold, and the Cas9 and Cas12a cleavage sites indicated by arrows. Nucleotide insertions and substitutions are shown in red; nucleotide deletions are indicated with—(WT, wild-type; −n, n nucleotides deleted; +n, n nucleotides inserted; NS, nucleotide substitution). The amino acid sequence of the translated Gag/capsid proteins is shown at the right with amino acid substitutions in red. The frameshift mutations result in several amino acid substitutions at the C-terminal end of the translated protein and a premature translation stop (indicated with *; X_n_, n non-Gag amino acids before translation stops). The 64-nt deletion observed in the Cas12a treated cells (TA clone 8) results in a truncated Gag protein with 86 capsid amino acids and 18 non-Gag amino acids at the C-terminal end.

When cells were treated with Cas9 mRNA, most of the proviral DNA was full length (96%), and only a small fraction was excised (3%) or inverted (1%) (Figure 3b). Sequencing of the full-length product revealed that nearly all fragments were wild-type, and only 1 out of 10 sequenced fragments carried a deletion at the gGag1 target site (Figure 4). The qPCR data (Figure 3b) were combined with the ratio between wild-type and mutated full-length fragments (Figure 4) to calculate the frequency of the different CRISPR-Cas induced products (Figure 3c). The observed high abundance of wild-type sequences agrees with the high level of proviral gene expression (Figure 2a–b), and the high production of infectious virus particles (Figure 2c) observed upon dox induction.

When cells were treated with Cas9 protein, excision and inversion products were observed at much higher frequencies (32% and 18%, respectively), while the fraction of full-length products was reduced (50%). Sequencing of the latter products demonstrated that the gGag1 target site was mutated in all sequenced fragments (Figure 4). We observed both nucleotide insertions and deletions, sometimes combined with nucleotide substitutions, around the Cas9 cleavage site. This mutational pattern very much resembles the indel pattern that we previously observed at gRNA target sites upon CRISPR-Cas9 attack of HIV in cells stably expressing Cas9 [11,16]. Most deletions and insertions at the gGag1 target site represent frameshift mutations that will interfere with translation of the downstream Gag/Pol sequences and abolish Gag/Pol protein production (Figure 4). The other mutations are triplet insertions that insert an additional amino acid in the protein, which will likely also inactivate or attenuate the virus as a highly conserved and essential CA domain in Gag is targeted. We only analyzed the gGag1 target site, but similar mutations are likely to occur at the simultaneously targeted gTatRev site, which also impairs Tat and Rev function and further blocks virus replication [11,16]. The combined qPCR and sequencing data indicate that all HIV proviral DNA is mutated after the 3rd Cas9 protein treatment (Figure 3c). The finding that target-site mutations occur more frequently than excision and especially inversion mimics what we observed upon stable CRISPR-Cas attack [18]. As not only excision and inversion of the sequences between the gGag1 and gTatRev1 target sites but likely also the indels at the gRNA target sites affect virus replication, these data suggest complete Cas9-induced HIV inactivation, resulting in the apparent cure of the infected cell culture. This is consistent with the fact that we could not detect any viral replication (Figure 2a), nor the production of infectious virus (Figure 2c), upon dox administration.

When cells were treated with Cas12a protein, most of the proviral DNA (94%) was found to be full length, and excisions (5%) and inversions (1%) were detected at much lower frequencies (Figure 3b). Sequencing of the crGag1 site in the full-length product demonstrated that 3 out of 10 sequenced fragments were wild type (Figure 4). Most of the fragments (7 out of 10) had nucleotide deletions around the Cas12a cleavage site, sometimes combined with nucleotide substitutions, but nucleotide insertions—as frequently observed upon Cas9 treatment—were not detected. This mutational pattern is consistent with the pattern previously described when targeting HIV with the CRISPR-Cas12a system in cells stably expressing Cas12a [19]. Most mutations at the crGag1 site are frameshift mutations in the Gag gene that will abolish the production of Gag/Pol proteins. The other mutations are 3 and 6 nt deletions that remove one or two amino acids in the important CA domain of Gag, which likely also inactivate or attenuate the virus. We did not sequence the crTatRev1 target site, and some of the proviruses with a wild-type crGag1 site may be mutated at the crTarRev1 site. However, it seems plausible that a fraction of the proviruses is not affected and has a wild-type sequence at both positions. Incomplete inactivation of the proviral DNA can explain the reduced level of viral gene expression and replication (Figure 3a–b), and the production of infectious virus (Figure 3c) observed upon dox administration.

## 4. Discussion

We tested whether HIV-infected cells can be cured by different transient CRISPR-Cas treatments in vitro. When cells that were latently infected with a replication-competent HIV strain were transfected three times with Cas9 protein and dual gRNAs targeting highly conserved Gag and Tat/Rev domains, we observed that all proviral genomes were mutated, viral gene expression was strongly reduced, and virus replication could no longer be reactivated. Similar treatment with Cas12a proteins and crRNAs resulted in the substantial but incomplete mutation of the proviral DNA, and viral gene expression and replication were found to be reduced but not prevented. Repeated transfection with Cas9 mRNA resulted in the mutation of only a small fraction of the provirus, which did not detectably reduce viral gene expression and replication. The observed differences in mutation efficiency are in agreement with previous observations. For example, when mammalian cells were transfected with different CRISPR-Cas9 formats for genome editing, Cas9 protein was found to be more efficient than Cas9 mRNA [35,36]. Possibly, the higher efficiency of the Cas9 protein is largely due to the fact that the protein was transfected into the nucleus (by nucleofection) as a pre-assembled Cas9 protein-gRNA ribonucleoprotein (RNP) complex that can immediately cleave the target DNA, whereas upon transfection of the mRNA, the mRNA first needs to be translated in the cytoplasm and the protein product has to complex with the gRNA and enter the nucleus before the DNA can be cleaved. Furthermore, upon microinjection of Cas9 or Cas12a protein combined with different gRNAs/crRNAs into zebrafish embryos, both Cas proteins resulted in high overall gene editing, but the editing efficiency measured with the Cas12a protein varied substantially for different crRNAs and was relatively low for several crRNAs [37]. We did not further investigate why the Cas9 mRNA and Cas12a protein treatments were less efficient than the Cas9 protein treatment in our experiments. Importantly, we demonstrated that repeated transient treatment with the Cas9 protein and dual gRNAs could completely prevent HIV replication. We previously reached permanent HIV inactivation in cells by stable transduction with lentiviral vectors that continuously produced the CRISPR-Cas antivirals, but lentiviral transduction and continuous CRISPR-Cas activity seem less suitable for in vivo application of this antiviral strategy. The observation that complete virus inactivation can also be reached by transient CRISPR-Cas treatment in vitro indicates that transient CRISPR-Cas delivery methods, such as ribonucleoprotein particles or virus-like particles [25,38,39], can be considered for in vivo application.

Several hurdles need to be overcome before this strategy can be successfully and safely applied in humans. Although a functional cure can perhaps be reached when the viral load is reduced below a certain level, complete virus inactivation may require that the CRISPR-Cas components are delivered to all HIV-infected cells. Currently available delivery methods may not reach a sufficient number of infected cells in vivo. Novel techniques may be needed that specifically target the HIV-infected reservoir cells, for example, facilitated by specific reservoir markers such as CD32a [40,41]. Immune responses against the non-human Cas protein may complicate such therapy, in particular when repeated treatments will be required [42,43]. Although the risk of Cas9 cleavage at non-target sites in the cellular DNA will likely be reduced by transient and titratable instead of continuous Cas activity, such off-target effects may still be an issue that requires further safety tests [44]. Another complicating factor for in vivo application is the high genetic diversity of HIV, as even a single nucleotide mismatch between the gRNA and the viral target can prevent Cas9 cleavage [32]. For most virus isolates, combining multiple gRNAs that target conserved and essential sequences will avoid this problem, while for some exotic HIV strains with divergent sequences, it may be necessary to design isolate-specific gRNAs/crRNAs [32,45,46,47].

Proviral DNA analysis upon repeated dual gRNA-Cas9 protein treatment revealed mutations at the gGag1 target site in 50% of the proviral DNA, whereas the fragment between the target sites had been excised or inverted in 32% and 18% of cases, respectively (Figure 3c). Similar frequencies for the different products were observed previously upon CRISPR-Cas9 attack of HIV in cells stably expressing Cas9 [18]. Upon repeated attack with dual crRNA-Cas12a protein, 66% of the proviral DNA was mutated at the crGag1 site, and the frequencies for excision and inversion were much lower (5% and 1%, respectively). Target site mutations are also the dominant product of dual crRNA/Cas12a activity in genome-editing applications [48,49,50]. These results indicate that Cas9 cleavage results more frequently in excision or inversion than Cas12a cleavage. This difference may be due to the different sequences targeted by Cas9 and Cas12a (because of different PAM requirements), as we previously observed different excision and inversion frequencies when using different gRNA pairs for Cas9 cleavage [18]. However, other Cas9 and Cas12 differences, such as the cleavage pattern (resulting in blunt-ended and sticky-ended DNA fragments, respectively), may have contributed to the observed frequency differences.

This study and our previous studies demonstrate that not only proviral DNA excision and inversion but also the more prevalent mutation of the target site can reduce viral protein production to very low or undetectable levels and permanently inactivate HIV replication. Nevertheless, excision and, to a lesser extent, inversion of large proviral DNA fragments seem a more favorite and definitive outcome of a CRISPR-Cas treatment. First, mutation may lead to escape variants. Although we and others [16,17] previously demonstrated that the odds for such escape could be significantly reduced by simultaneous targeting two or more carefully selected viral sequences, it is obvious that excision or inversion will completely abolish virus escape. Second, excision and inversion will completely block the expression of encoded viral proteins, while mutations may not prevent low-level protein production or could result in the production of aberrant proteins. Cells expressing such viral proteins can be eliminated by cytotoxic T cells [51,52,53] but may increase the level of immune activation and inflammation [54,55]. Several studies aimed at the excision of large proviral DNA fragments [56,57,58,59,60,61,62,63,64], but from our quantitative analyses, it is clear that excision is far from complete, and further research will be needed to increase the excision frequency.

## Data Availability

Not applicable.

## Supplementary Materials

The following are available online at https://www.mdpi.com/article/10.3390/v13122461/s1, Figure S1: Phenotype analysis of HIV-rtTA-GFP infected cells after the 1st and 2nd Cas treatment.

## References

[1] Siliciano J.D., Kajdas J., Finzi D., Quinn T.C., Chadwick K., Margolick J.B., Kovacs C., Gange S., Siliciano R.F. (2003). Long-term follow-up studies confirm the stability of the latent reservoir for HIV-1 in resting CD4+ T cells. Nat. Med..

[2] Blankson J.N., Persaud D., Siliciano R.F. (2002). The Challenge of Viral Reservoirs in HIV-1 Infection. Annu. Rev. Med..

[3] Chun T.-W., Stuyver L., Mizell S.B., Ehler L.A., Mican J.A.M., Baseler M., Lloyd A., Nowak M.A., Fauci A.S. (1997). Presence of an inducible HIV-1 latent reservoir during highly active antiretroviral therapy. Proc. Natl. Acad. Sci. USA.

[4] Davey R.T., Bhat N., Yoder C., Chun T.-W., Metcalf J.A., Dewar R., Natarajan V., Lempicki R., Adelsberger J.W., Miller K.D. (1999). HIV-1 and T cell dynamics after interruption of highly active antiretroviral therapy (HAART) in patients with a history of sustained viral suppression. Proc. Natl. Acad. Sci. USA.

[5] Colby D.J., Trautmann L., Pinyakorn S., Leyre L., Pagliuzza A., Kroon E., Rolland M., Takata H., Buranapraditkun S., Intasan J. (2018). Rapid HIV RNA rebound after antiretroviral treatment interruption in persons durably suppressed in Fiebig I acute HIV infection. Nat. Med..

[6] Wang G., Zhao N., Berkhout B., Das A.T. (2018). CRISPR-Cas based antiviral strategies against HIV-1. Virus Res..

[7] Atkins A.J., Allen A.G., Dampier W., Haddad E.K., Nonnemacher M.R., Wigdahl B. (2021). HIV-1 cure strategies: Why CRISPR?. Expert Opin. Biol. Ther..

[8] Jinek M., Chylinski K., Fonfara I., Hauer M., Doudna J.A., Charpentier E. (2012). A Programmable dual-RNA-guided DNA endonuclease in adaptive bacterial immunity. Science.

[9] Shen M.W., Arbab M., Hsu J.Y., Worstell D., Culbertson S.J., Krabbe O., Cassa C.A., Liu D.R., Gifford D.K., Sherwood R.I. (2018). Predictable and precise template-free CRISPR editing of pathogenic variants. Nat. Cell Biol..

[10] Allen F., Crepaldi L., Alsinet C., Strong A.J., Kleshchevnikov V., De Angeli P., Páleníková P., Khodak A., Kiselev V., Kosicki M. (2019). Predicting the mutations generated by repair of Cas9-induced double-strand breaks. Nat. Biotechnol..

[11] Wang G., Zhao N., Berkhout B., Das A.T. (2016). CRISPR-Cas9 Can Inhibit HIV-1 Replication but NHEJ Repair Facilitates Virus Escape. Mol. Ther..

[12] Yoder K.E., Bundschuh R. (2016). Host Double Strand Break Repair Generates HIV-1 Strains Resistant to CRISPR/Cas9. Sci. Rep..

[13] Ueda S., Ebina H., Kanemura Y., Misawa N., Koyanagi Y. (2016). Anti-HIV-1 potency of the CRISPR/Cas9 system insufficient to fully inhibit viral replication. Microbiol. Immunol..

[14] Wang Z., Pan Q., Gendron P., Zhu W., Guo F., Cen S., Wainberg M.A., Liang C. (2016). CRISPR/Cas9-Derived Mutations Both Inhibit HIV-1 Replication and Accelerate Viral Escape. Cell Rep..

[15] Liang C., Wainberg M.A., Das A.T., Berkhout B. (2016). CRISPR/Cas9: A double-edged sword when used to combat HIV infection. Retrovirology.

[16] Wang G., Zhao N., Berkhout B., Das A.T. (2016). A Combinatorial CRISPR-Cas9 Attack on HIV-1 DNA Extinguishes All Infectious Provirus in Infected T Cell Cultures. Cell Rep..

[17] Lebbink R.J., De Jong D.C.M., Wolters F., Kruse E.M., Van Ham P.M., Wiertz E.J.H.J., Nijhuis M. (2017). A combinational CRISPR/Cas9 gene-editing approach can halt HIV replication and prevent viral escape. Sci. Rep..

[18] Binda C.S., Klaver B., Berkhout B., Das A.T. (2020). CRISPR-Cas9 Dual-gRNA Attack Causes Mutation, Excision and Inversion of the HIV-1 Proviral DNA. Viruses.

[19] Gao Z., Fan M., Das A.T., Herrera-Carrillo E., Berkhout B. (2020). Extinction of all infectious HIV in cell culture by the CRISPR-Cas12a system with only a single crRNA. Nucleic Acids Res..

[20] Liu Z., Liang J., Chen S., Wang K., Liu X., Liu B., Xia Y., Guo M., Zhang X., Sun G. (2020). Genome editing of CCR5 by AsCpf1 renders CD4+T cells resistance to HIV-1 infection. Cell Biosci..

[21] Zetsche B., Gootenberg J.S., Abudayyeh O.O., Slaymaker I.M., Makarova K.S., Essletzbichler P., Volz S.E., Joung J., van der Oost J., Regev A. (2015). Cpf1 Is a Single RNA-Guided Endonuclease of a Class 2 CRISPR-Cas System. Cell.

[22] Fagerlund R.D., Staals R.H.J., Fineran P.C. (2015). The Cpf1 CRISPR-Cas protein expands genome-editing tools. Genome Biol..

[23] Kim D., Kim J., Hur J.K., Been K.W., Yoon S.-H., Kim J.-S. (2016). Genome-wide analysis reveals specificities of Cpf1 endonucleases in human cells. Nat. Biotechnol..

[24] Gao Z., Herrera-Carrillo E., Berkhout B. (2019). A Single H1 Promoter Can Drive Both Guide RNA and Endonuclease Expression in the CRISPR-Cas9 System. Mol. Ther. Nucleic Acids.

[25] Campbell L., Coke L.M., Richie C.T., Fortuno L.V., Park A.Y., Harvey B.K. (2019). Gesicle-Mediated Delivery of CRISPR/Cas9 Ribonucleoprotein Complex for Inactivating the HIV Provirus. Mol. Ther..

[26] Peden K., Emerman M., Montagnier L. (1991). Changes in growth properties on passage in tissue culture of viruses derived from infectious molecular clones of HIV-1LAI, HIV-1MAL, and HIV-1ELI. Virology.

[27] Das A.T., Berkhout B. (2016). Conditionally replicating HIV and SIV variants. Virus Res..

[28] Das A.T., Zhou X., Vink M., Klaver B., Verhoef K., Marzio G., Berkhout B. (2004). Viral evolution as a tool to improve the tet-racycline-regulated gene expression system. J. Biol. Chem..

[29] Das A.T., Tenenbaum L., Berkhout B. (2016). Tet-On Systems For Doxycycline-inducible Gene Expression. Curr. Gene Ther..

[30] Smith S.D., Shatsky M., Cohen P.S., Warnke R., Link M.P., E Glader B. (1984). Monoclonal antibody and enzymatic profiles of human malignant T-lymphoid cells and derived cell lines. Cancer Res..

[31] E Jeeninga R., Jan B., Berg H.V.D., Berkhout B. (2006). Construction of doxycyline-dependent mini-HIV-1 variants for the development of a virotherapy against leukemias. Retrovirology.

[32] Darcis G., Binda C.S., Klaver B., Herrera-Carrillo E., Berkhout B., Das A.T. (2019). The Impact of HIV-1 Genetic Diversity on CRISPR-Cas9 Antiviral Activity and Viral Escape. Viruses.

[33] Berkhout B., Verhoef K., Marzio G., Klaver B., Vink M., Zhou X., Das A. (2002). Conditional Virus Replication as an Approach to a Safe Live Attenuated Human Immunodeficiency Virus Vaccine. J. NeuroVirology.

[34] Das A.T., Baldwin C.E., Vink M., Berkhout B. (2005). Improving the Safety of a Conditional-Live Human Immunodeficiency Virus Type 1 Vaccine by Controlling both Gene Expression and Cell Entry. J. Virol..

[35] Kouranova E., Forbes K., Zhao G., Warren J., Bartels A., Wu Y., Cui X. (2016). CRISPRs for Optimal Targeting: Delivery of CRISPR Components as DNA, RNA, and Protein into Cultured Cells and Single-Cell Embryos. Hum. Gene Ther..

[36] Liang X., Potter J., Kumar S., Zou Y., Quintanilla R., Sridharan M., Carte J., Chen W., Roark N., Ranganathan S. (2015). Rapid and highly efficient mammalian cell engineering via Cas9 protein transfection. J. Biotechnol..

[37] Meshalkina D., Glushchenko A., Kysil E., Mizgirev I., Frolov A. (2020). SpCas9- and LbCas12a-Mediated DNA Editing Produce Different Gene Knockout Outcomes in Zebrafish Embryos. Genes.

[38] Choi J.G., Dang Y., Abraham S., Ma H., Zhang J., Guo H., Cai Y., Mikkelsen J.G., Wu H., Shankar P. (2016). Lentivirus pre-packed with Cas9 protein for safer gene editing. Gene Ther..

[39] Montagna C., Petris G., Casini A., Maule G., Franceschini G.M., Zanella I., Conti L., Arnoldi F., Burrone O.R., Zentilin L. (2018). VSV-G-Enveloped Vesicles for Traceless Delivery of CRISPR-Cas9. Mol. Ther. Nucleic Acids.

[40] Darcis G., Kootstra N.A., Hooibrink B., van Montfort T., Maurer I., Groen K., Jurriaans S., Bakker M., van Lint C., Berkhout B. (2020). CD32^+^CD4^+^ T Cells Are Highly Enriched for HIV DNA and Can Support Transcriptional Latency. Cell Rep..

[41] Descours B., Petitjean G., López-Zaragoza J.-L., Bruel T., Raffel R., Psomas C., Reynes C.P.J., Lacabaratz C., Levy Y., Schwartz O. (2017). CD32a is a marker of a CD4 T-cell HIV reservoir harbouring replication-competent proviruses. Nat. Cell Biol..

[42] Wang D., Mou H., Li S., Li Y., Hough S., Tran K., Li J., Yin H., Anderson D.G., Sontheimer E.J. (2015). Adenovirus-Mediated Somatic Genome Editing of Pten by CRISPR/Cas9 in Mouse Liver in Spite of Cas9-Specific Immune Responses. Hum. Gene Ther..

[43] Chew W.L., Tabebordbar M., Cheng J.K., Mali P., Wu E.Y., Ng A.H., Zhu K., Wagers A.J., Church G.M. (2016). A multifunctional AAV-CRISPR-Cas9 and its host response. Nat. Methods.

[44] Atkins A., Chung C.-H., Allen A.G., Dampier W., Gurrola T.E., Sariyer I.K., Nonnemacher M.R., Wigdahl B. (2021). Off-Target Analysis in Gene Editing and Applications for Clinical Translation of CRISPR/Cas9 in HIV-1 Therapy. Front. Genome Ed..

[45] Dampier W., Sullivan N., Mell J.C., Pirrone V., Ehrlich G.D., Chung C.-H., Allen A.G., DeSimone M., Zhong W., Kercher K. (2018). Broad-Spectrum and Personalized Guide RNAs for CRISPR/Cas9 HIV-1 Therapeutics. AIDS Res. Hum. Retroviruses.

[46] Sullivan N., Dampier W., Chung C.-H., Allen A.G., Atkins A., Pirrone V., Homan G., Passic S., Williams J., Zhong W. (2019). Novel gRNA design pipeline to develop broad-spectrum CRISPR/Cas9 gRNAs for safe targeting of the HIV-1 quasispecies in patients. Sci. Rep..

[47] Roychoudhury P., Feelixge H.D.S., Reeves D., Mayer B.T., Stone D., Schiffer J.T., Jerome K.R. (2018). Viral diversity is an obligate consideration in CRISPR/Cas9 designs for targeting the HIV reservoir. BMC Biol..

[48] Kim Y., Cheong S.-A., Lee J.G., Lee S.-W., Lee M.S., Baek I.-J., Sung Y.H. (2016). Generation of knockout mice by Cpf1-mediated gene targeting. Nat. Biotechnol..

[49] Sun H., Li F., Liu J., Yang F., Zeng Z., Lv X., Tu M., Liu Y., Ge X., Liu C. (2018). A Single Multiplex crRNA Array for FnCpf1-Mediated Human Genome Editing. Mol. Ther..

[50] Duan K., Cheng Y., Ji J., Wang C., Wei Y., Wang Y. (2021). Large chromosomal segment deletions by CRISPR/LbCpf1-mediated multiplex gene editing in soybean. J. Integr. Plant Biol..

[51] Huang S.-H., Ren Y., Thomas A.S., Chan D., Mueller S., Ward A., Patel S., Bollard C.M., Cruz C.R., Karandish S. (2018). Latent HIV reservoirs exhibit inherent resistance to elimination by CD8+ T cells. J. Clin. Investig..

[52] Imamichi H., Dewar R.L., Adelsberger J.W., Rehm C.A., O’Doherty U., Paxinos E.E., Fauci A.S., Lane H.C. (2016). Defective HIV-1 proviruses produce novel protein-coding RNA species in HIV-infected patients on combination antiretroviral therapy. Proc. Natl. Acad. Sci. USA.

[53] Pollack R.A., Jones R.B., Pertea M., Bruner K.M., Martin A.R., Thomas A., Capoferri A.A., Beg S.A., Huang S.-H., Karandish S. (2017). Defective HIV-1 Proviruses Are Expressed and Can Be Recognized by Cytotoxic T Lymphocytes, which Shape the Proviral Landscape. Cell Host Microbe.

[54] Hatano H., Jain V., Hunt P.W., Lee T.-H., Sinclair E., Do T.D., Hoh R., Martin J.N., McCune J.M., Hecht F. (2013). Cell-Based Measures of Viral Persistence Are Associated With Immune Activation and Programmed Cell Death Protein 1 (PD-1)–Expressing CD4+ T cells. J. Infect. Dis..

[55] Deeks S.G., Tracy R., Douek D.C. (2013). Systemic Effects of Inflammation on Health during Chronic HIV Infection. Immunity.

[56] Ebina H., Misawa N., Kanemura Y., Koyanagi Y. (2013). Harnessing the CRISPR/Cas9 system to disrupt latent HIV-1 provirus. Sci. Rep..

[57] Hu W., Kaminski R., Yang F., Zhang Y., Cosentino L., Li F., Luo B., Alvarez-Carbonell D., Garcia-Mesa Y., Karn J. (2014). RNA-directed gene editing specifically eradicates latent and prevents new HIV-1 infection. Proc. Natl. Acad. Sci. USA.

[58] Liao H.-K., Gu Y., Diaz A., Marlett J.M., Takahashi Y., Li M., Suzuki K., Xu R., Hishida T., Chang C.J. (2015). Use of the CRISPR/Cas9 system as an intracellular defense against HIV-1 infection in human cells. Nat. Commun..

[59] Kaminski R., Chen Y., Fischer T., Tedaldi E., Napoli A., Zhang Y., Karn J., Hu W., Khalili K. (2016). Elimination of HIV-1 Genomes from Human T-lymphoid Cells by CRISPR/Cas9 Gene Editing. Sci. Rep..

[60] Kaminski R., Bella R., Yin C., Otte J., Ferrante P., E Gendelman H., Li H., Booze R., Gordon J., Hu W. (2016). Excision of HIV-1 DNA by gene editing: A proof-of-concept in vivo study. Gene Ther..

[61] Ran F.A., Cong L., Yan W.X., Scott D.A., Gootenberg J., Kriz A.J., Zetsche B., Shalem O., Wu X., Makarova K.S. (2015). In vivo genome editing using Staphylococcus aureus Cas9. Nat. Cell Biol..

[62] Yin C., Zhang T., Li F., Yang F., Putatunda R., Young W.-B., Khalili K., Hu W., Zhang Y. (2016). Functional screening of guide RNAs targeting the regulatory and structural HIV-1 viral genome for a cure of AIDS. AIDS.

[63] Dampier W., Nonnemacher M.R., Sullivan N.T., Jacobson J.M., Wigdahl B. (2014). HIV Excision Utilizing CRISPR/Cas9 Technology: Attacking the Proviral Quasispecies in Reservoirs to Achieve a Cure. MOJ Immunol..

[64] Yin C., Zhang T., Qu X., Zhang Y., Putatunda R., Xiao X., Li F., Xiao W., Zhao H., Dai S. (2017). In Vivo Excision of HIV-1 Provirus by saCas9 and Multiplex Single-Guide RNAs in Animal Models. Mol. Ther..

