# Supplementary material for: Transient CRISPR-Cas Treatment Can Prevent Reactivation of HIV-1 Replication in a Latently Infected T-Cell Line
Source: Viruses. 2021 Dec 8;13(12):2461. doi: 10.3390/v13122461 (PMC8705111; doi:10.3390/v13122461)
Supplement: Supplementary file 1 [file viruses-13-02461-s001.zip › viruses-1468911-supplementary.pdf]

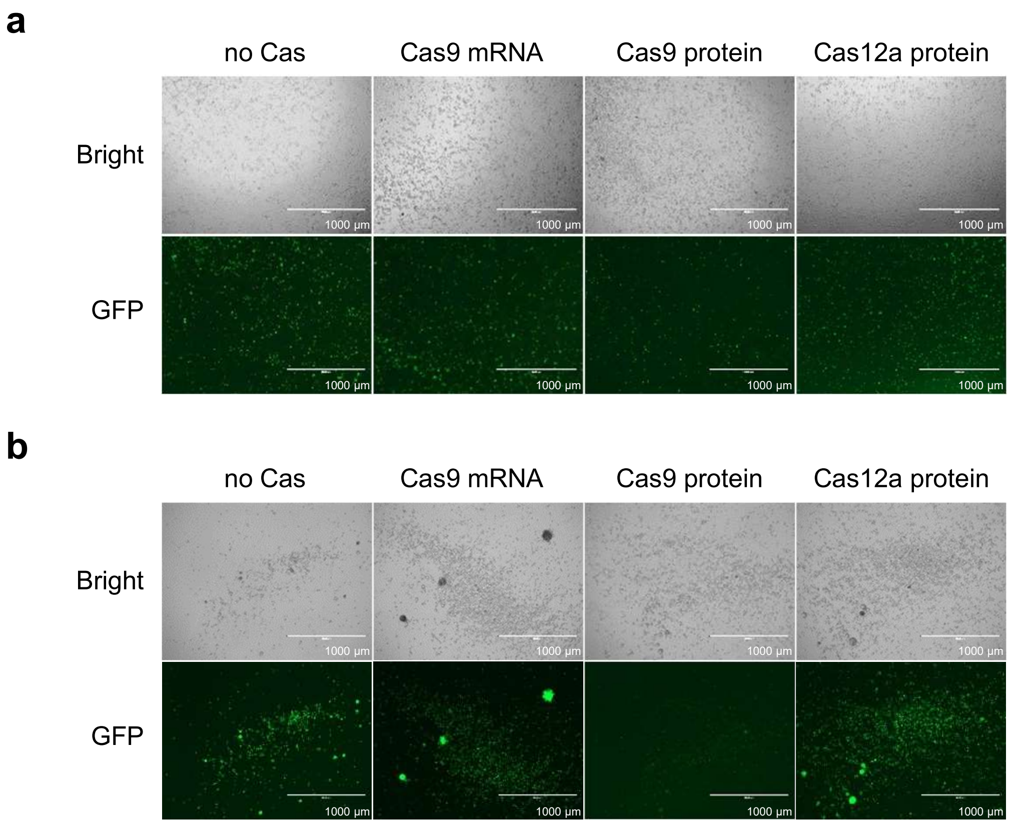

Figure S1. Phenotype analysis of HIV-rtTA-GFP infected cells after the 1st (a) and 2nd (b) Cas treatment. Control (no Cas) and CRISPR-Cas treated cells were cultured with dox for 3 days and virus-induced syncytia formation and GFP production was analyzed by bright field and fluorescence microscopy.

Supplementary Figure S1
